# Craniofacial morphology in Obstructive Sleep Apnea patients

**DOI:** 10.4317/jced.61104

**Published:** 2023-12-01

**Authors:** Domenico Ciavarella, Mauro Lorusso, Alessandra Campobasso, Angela-Pia Cazzolla, Graziano Montaruli, Giuseppe Burlon, Eleonora Lo Muzio, Michele Laurenziello, Michele Tepedino

**Affiliations:** 1 Department of Clinical and Experimental Medicine, Dental School of Foggia, University of Foggia, Foggia, Italy; 2 Department of Translational Medicine and for Romagna, School of Orthodontics, University of Ferrara, Ferrara, Italy; 3 Department of Biotechnological and Applied Clinical Sciences, Dental School of L’Aquila, University of L’Aquila, L’Aquila, Italy

## Abstract

**Background:**

To evaluate the correlation between cephalometric skeletal parameters and Obstructive Sleep Apnea syndrome (OSAs) severity, in adult patients with OSAs.

**Material and Methods:**

One hundred patients (94 males,6 females mean age 59,3) with diagnosis of OSAs were retrospectively enrolled. Each patient received Home Sleep Apnea Testing (HSAT) and latero-lateral radiograph. Eight cephalometric parameters (cranial deflection angle, saddle angle, articular angle, divergence angle, cranial base angle, skull base length, mandibular length, maxilla length) were analyzed and then related to Apnea/Hypopnea Index (AHI) and to the Oxygen Desaturation Index (ODI), recorded by HSAT. A Spearman’s rho correlation test between cephalometric measurements and HSAT indices was performed. Statistical significance was set at *p*< 0.05.

**Results:**

A negative statistically significant correlation was found between mandibular length (Condilion-Gnathion distance) and AHI (rho= -0,2022; *p*<0,05) and between maxilla length (Ans-Pns) and AHI (rho= -0,2984; *p*<0,01) and ODI (rho= -0,2443; *p*<0,05). A statistically significant correlation was also observed between the divergence angle (S-N^Go-Me) and AHI (rho=0,2263; *p*<0,05) and between cranial deflection angle (Fh^NBa) and AHI (rho=0,2212; *p*<0,05) and ODI (rho=0,1970; *p*<0,05).

**Conclusions:**

The OSAs severity may be related to certain predisposing features in craniofacial morphology, such as maxillary and mandibular length, divergence and cranial deflection.

** Key words:**OSAs, Home Sleep Apnea Testing, AHI, ODI, Cephalometry, Airway.

## Introduction

Sleep disorders are common health problems in the population, with prevalence rates ranging from approximately 18% in Europe to 23% in the United States among the working population (1). The risks connected to disordered sleep represent a real emergency for health, work, and therapeutic management (2). The Obstructive Sleep Apnea syndrome (OSAs) is a sleep-related breathing disorder characterized by apnea or hypopnea events followed by transient awakenings that lead to the restoration of airway patency, obstruction of the upper respiratory tract, decreased blood oxygen saturation, fragmented sleep, and excessive daytime sleepiness (2). Although it is more common in middle-aged males, OSAs can occur in any age group, affecting 2-4% of the adult population (3). On average, one in five adults has moderate OSAs, and one in fifteen has moderate or severe OSAs (3). The prevalence of OSAs is approximately 22% in men and 17% in women. The gender distribution follows a ratio of 2:1, with men outnumbering women (4). This gender distribution is likely attributed to differing hormonal effects that lead to increased collapsibility of upper airway muscles, distinct body fat distribution, and varied anatomy. Hormonal effects play a significant role in the pathogenesis of OSAs, especially in postmenopausal women compared to those in the premenopausal stage. Unfortunately, the exact role of hormones in OSA pathogenesis remains unclear (5). Several risk factors are involved in the occurrence of OSAs, including male gender, middle age, ethnicity, smoking, alcohol consumption, obesity, and craniofacial morphology of both soft and hard tissues, which can alter the size and shape of the upper airways (6). The most common symptoms of OSAs are apneas, which result from the narrowing and intermittent collapse of the upper airways during sleep (7). The American Academy of Sleep Medicine (AASM) has defined apnea as a complete airway obstruction characterized by the intermittent interruption of respiratory flow, with a reduction of more than 90% during sleep, lasting for at least 10 seconds, associated with oxyhemoglobin desaturation and persistence of thoracic and/or abdominal respiratory movements (8). Another frequent symptom of OSAs is chronic snoring, usually reported by the patient’s partner (7). Although it has low diagnostic value, snoring affects over 95% of adult patients, approximately 25-30% of women, and 40-45% of men (9). Daytime sleepiness is also commonly associated with OSAs, resulting from the sleep fragmentation caused by electroencephalographic awakenings that interrupt oxygen desaturation (9). In fact, literature reports that OSAs can significantly increase the risk of workplace injuries. It induces excessive daytime sleepiness, decline in cognitive functions (such as difficulty in concentration, attention, and memory, as well as slowing of reflexes and ideation), and impaired neuro-motor coordination (10). Cardiovascular disease is associated with OSAs, with the primary factors contributing to this correlation being sympathetic activation, oxidative stress, and systemic inflammation. OSAs stands out as an independent risk factor for conditions such as hypertension, coronary artery disease, heart failure, cardiovascular and cerebrovascular disease, and atrial fibrillation (11-13) Additionally, OSAs is connected to numerous metabolic complications, including type 2 diabetes mellitus. The prevalence of type 2 diabetes mellitus among OSAs patients surpasses that of the general population (14). The evaluation of OSA is carried out using various questionnaires. One type of questionnaire focuses on assessing daytime sleepiness and health-related quality of life (HRQoL) (15). The Epworth Sleepiness Scale (ESS), the Stop Bang questionnaire, and the Berlin questionnaire are among the main tools utilized to evaluate daytime sleepiness (16). Many tests are available for the diagnosis of OSAs, and the gold standard is level I testing by overnight laboratory polysomnography (PSG), which monitors sleep status, breathing, electrocardiogram, leg movements, oximetry, and snoring (8). Although PSG is considered the gold standard test for diagnosing OSAs, it does not offer precise localization of the site of airflow obstruction. Nasal endoscopy is a valuable tool for assessing the level, degree, and shape of upper airway obstruction. Typically, this procedure is conducted in an operating room. Various imaging techniques, such as X-ray cephalometry, sleep videofluoroscopy, computed tomography (CT) scanning, and magnetic resonance imaging (MRI), have been utilized to pinpoint the site of obstruction and identify other structural abnormalities.

However, given the ever-increasing number of sleep disorders, sleep laboratories and their diagnostic tools are in high demand. Alternative methods are proposed in the literature for the screening and diagnosis of OSAs, such as Home Sleep Apnea Testing (HSAT) (17). The HSAT is a level III diagnostic tool that can be performed in home settings without sleep technologists, representing a less expensive diagnostic option (17). Accurate diagnosis holds immense importance, particularly within EU countries, where compliance with Commission Directive 2014/85/EU mandates testing for OSAs before issuing or renewing a driver’s license. Individuals with moderate or severe OSAs who are receiving treatment are also required to undergo periodic medical evaluations. Various therapeutic approaches are available for the treatment of OSAs. Continuous positive airway pressure (CPAP) is the first-line treatment for patients with obstructive sleep apnea. This non-invasive approach aims to maintain airway patency by delivering a consistent airway pressure. Alternatively, other methods, such as oral appliances, are employed, particularly for individuals who have difficulty tolerating a CPAP mask. Surgery, on the other hand, is reserved for cases involving anatomical obstructions requiring correction (18).

The pathogenesis of OSAs is multifactorial (19,20), although one of the most frequent cause is a reduction in the contraction capacity and force of the pharyngeal dilator muscles, attributed to incoordination of muscle activity and respiratory effort (21). Other pathogenetic factors include soft palate hypertrophy or hyperextension, macroglossia, tonsillar and pharyngeal hypertrophy, and craniofacial morphology alteration (22-24). Although previous studies have reported that cervical, hyoid, and mandibular cephalometric positions may influence the severity of OSAs (25,26), the influence of the craniofacial morphology on the development of OSAs is still controversial (24).

The aim of this study is to evaluate the correlations between cephalometric skeletal parameters and the severity of OSAs, in order to identify any predisposing facial morphological characteristics.

## Material and Methods

This study was conducted following the Strengthening The Reporting of OBservational Studies in Epidemiology (STROBE) guidelines for observational studies(27).

One hundred Caucasian patients (94 males, 6 females) with a mean age of 59.3 were retrospectively enrolled in the present study. The patients were treated at the Department of Orthodontics, University of Foggia, Italy, in chronological order from March 2018 to November 2021. All the procedures of this research protocol have adhered to the Declaration of Helsinki and have been approved by the Ethics Committee of the University of Foggia. The records were retrieved retrospectively, analyzed anonymously, and patients signed a written informed consent. A power analysis (G*Power 3.1.9.2, Franz Faul, Universitat Kiel, Germany) revealed that to detect a large effect size of 0.5 (28) with a linear multiple regression, considering an effect size of 0.15, α error prob of 0.05 and a power (1-β error prob) of 0.95, 74 subjects would be needed.

The inclusion criteria were as follows: age between 51 and 70, positive diagnosis of Osas, no maxillofacial and airway surgery, body mass index < 30 kg/m2, teleradiographs performed with the same cephalostat, no treatment with CPAP and no TMJ disease. The exclusion criteria were as follows: age <51 and >71, body mass index > 30kg/m2, smoking habit, cardiovascular or pulmonary disease, neurological disorders and previous cervical trauma. For each patient, Home Sleep Apnea Testing (HSAT) and lateral cephalogram were performed. Patient’s anthropometric and clinical data are listed in Table 1.

**Table 1:**
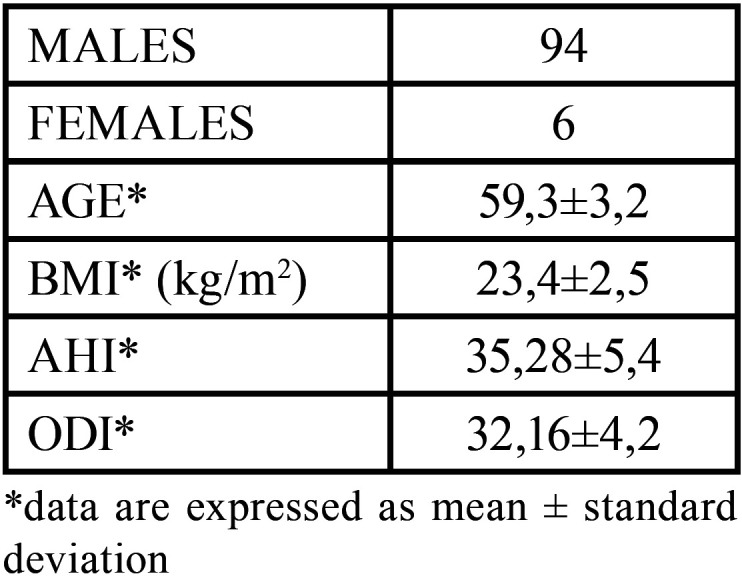
Patient’s anthropometric and clinical data.

In order to exclude temporomandibular disorders, each patient included in the study underwent a comprehensive gnathological examination, which included a focused questionnaire to assess daily habits, palpation of muscles and joints, evaluation of endfeel, auscultation, and assessment of the degree of mouth opening to closing.

-HSAT evaluation.

The data from the HSAT recordings were used for the diagnosis of OSAs according to the American Academy of Sleep Medicine (AASM) criteria from 2012 (29). A positive diagnosis of OSAs was made using the AHI index, with a value greater than 5 events per hour. The Apnea-Hypopnea Index (AHI) and the Oxygen Desaturation Index (ODI), indicating OSAs severity, were analyzed. The Apnea-Hypopnea Index (AHI) indicates the number of apnea and hypopnea events per hour of sleep. It defines the severity of Obstructive Sleep Apnea (OSA) as follows: mild (5-14.9), moderate (15-30), and severe (>30). An AHI of <5 is considered normal. The ODI index quantifies the frequency of oxygen desaturation by 4% per hour during sleep. The following signals were recorded during the HSAT: nasal pressure, rib cage and abdominal movement by respiratory inductance plethysmography, snoring, body position, heart rate and oxygen saturation. A trained sleep technologist demonstrated to participants how to apply the sensors at the sleep center. A successful HSAT required at least 3 hours of recording containing oxygen saturation and at least 1 of the respiratory signals. All HSATs provided by patients had been performed correctly and were used for the study.

-Cephalometric analysis.

Lateral head films (Gendex GXDP-700) were taken with the patient positioned in a cephalostat, maintaining centric occlusion, ensuring adequate visualization of reference structures, and avoiding appreciable head rotation, while keeping the Frankfurt horizontal plane parallel to the ground. The natural head position was established and secured using the cephalostat. All the lateral radiographs were captured by the same technician using the same machine in the same radiology department.

Several variables for skeletal tissue morphology were selected, including maxilla and mandible length, mandible projection relative to the skull base, skull base inclination, and length.

**Figure 1:**
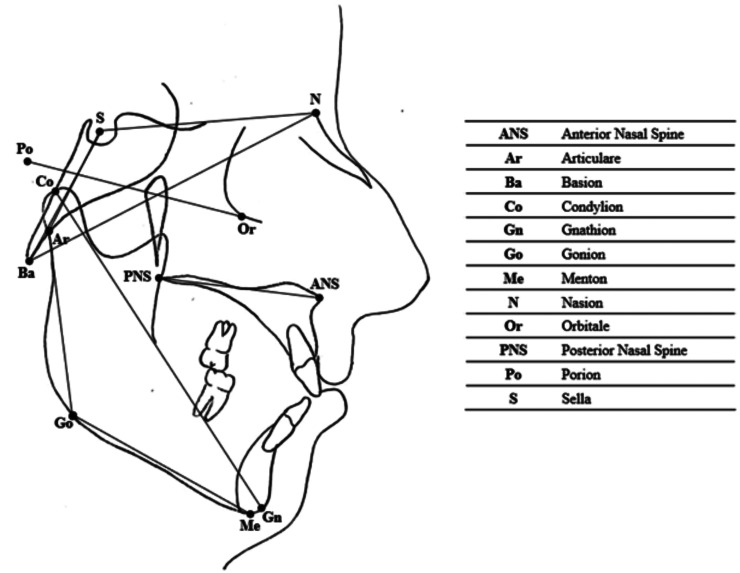
Cephalometric landmarks and reference lines.

A cephalometric analysis was performed on lateral cephalograms. The following cephalometric skeletal variables were analysed: Fh^N-Ba, S-N^Go-Me, S^N^Ba, S^Ar^Go, S^N^Ar, S-Ar, Co-Gn and Ans-Pns. The landmarks and reference lines used in the cephalometric analysis were presented in Figure 1 and described in Table 2.

**Table 2:**
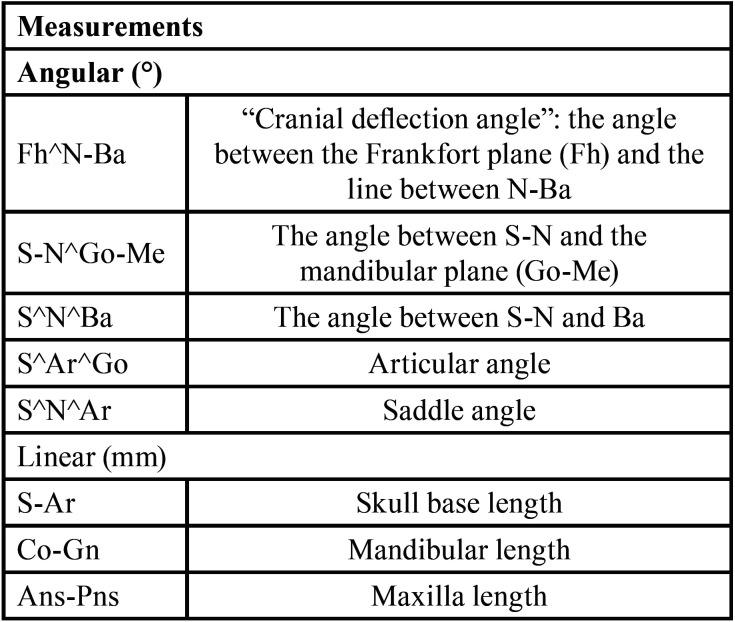
Description of cephalometric measurements.

To reduce the error of the method, the cephalometric analyses were performed by a trained examiner and all measurements were conducted twice by the same operator.

-Statistical analysis

Shapiro-Wilk normality test was performed to assess data distribution (Table 3). For data that did not follow a Gaussian distribution model, the correlation coefficient (Rho) for Spearman ranks was calculated to evaluate the presence of a statistically significant correlation between skeletal cephalometric parameters and HSAT indices. Pearson’s test was calculated for normally distributed data (Table 4). Statistical significance was set at *p* < 0.05 (Table 4). Statistical analysis was performed using SPSS software (SPSS for Windows, Version 15.0, Chicago, SPSS Inc).

**Table 3:**
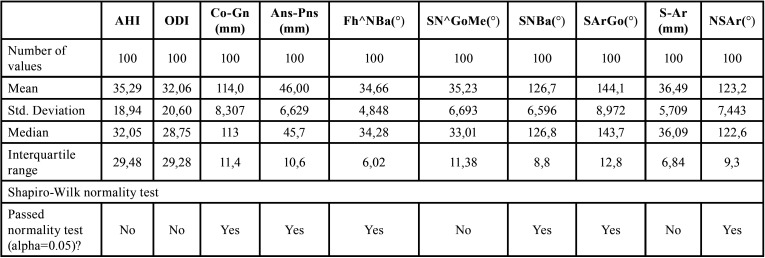
Descriptive statistics and Shapiro-Wilk normality test.

**Table 4:**
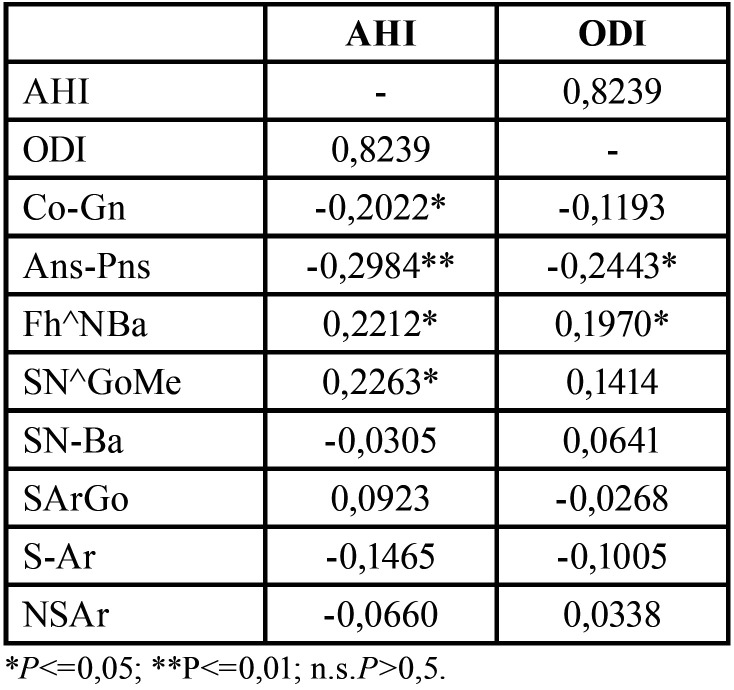
Spearman’s rho correlation test and Pearson’s test.

The random errors of each measurements was calculated using Dahlberg’s formula (S =∑ d 2 / 2N ), where d is the difference between the first and second measurements and N the number of radiographs evaluated(30,31) The random error ranged between 0.15 and 0.29 mm for linear measurements and between 0.18 and 0.23 degrees for angular measurements.

## Results

Statistical analysis results (Table 4) showed a negative significant correlation between mandibular length (Co-Gn) and AHI (rho= -0,2022; *p*<0,05), and between maxillary length (Ans-Pns) and AHI (rho= -0,2984; *p*<0,01) and ODI (rho= -0,2443; *p*<0,05). A statistically significant correlation was also observed between the divergence angle (S-N^Go-Me) and AHI (rho=0,2263; *p*<0,05), and between cranial deflection angle (Fh^NBa) and AHI (rho=0,2212; *p*<0,05) and ODI (rho=0,1970; *p*<0,05). No additional significant correlations were observed.

## Discussion

OSAs has a multifactorial and complex aetiology which has also been related to the soft tissues morphology and to the jaws skeletal alterations (32). However, there are only few studies in the literature that analyze the correlation between cephalometric parameters and OSAs severity (33-35). Therefore, the relationship between craniofacial anatomical variables and obstructive indices is actually still unclear (33).

In the present study, the cephalometric parameters of the main craniofacial structures have been related to OSAs indices, to evaluate whether alterations in the craniofacial skeletal structures may influence OSAs severity. In this study, only patients with normal BMI (body mass index) were evaluated to avoid any possible bias, because of the strong relationship between OSAs and BMI variations (37). Obesity represents a significant hazard for OSAs due to the enlargement of soft tissue structures within and encircling the airway, thus substantially contributing to the constriction of the pharyngeal airway. An excess of fat deposition has also been observed beneath the mandible and in the tongue, soft palate, and uvula (38).

The results obtained from this study showed a negative correlation between mandibular length (Co-Gn distance) and AHI (rho=-0,2022;*p*=0,043) and between maxilla length (Ans-Pns) and AHI (rho=-0,2984;*p*=0,002) and ODI (rho=-0,2443;*p*=0,014), and a significant correlation between the divergence angle (SN^GoMe) and AHI (rho=0,2263;*p*=0,023) and between cranial deflection angle (Fh^NBa) and AHI (rho=0,2212;*p*=0,026) and ODI (rho=0,1970;*p*=0,049). The significant correlation between Co-Gn distance and AHI suggested that, as mandibular length decreases, the number of obstructive events and of the apneas increases: therefore, it was suggested that the mandibular length could (directly or indirectly) modify the air passage through the upper airway.

In literature, the relation between OSAs and mandibular length is still controversial and most studies on this topic are case-controls, using the mandibular length as a comparative variable between OSAs patients and healthy control groups (39). The meta-analysis of Miles’ *et al*. (40) reported that only three studies demonstrated a reduction in mandibular length (measured as Go-Gn distance) among OSAs patients compared to controls. Gungor *et al*. (41) also observed a slight decrease in mandibular length (Co-Gn distance) in OSAs patients compared to the control group, but this difference was not statistically significant. Banhiran *et al*. (42) showed no significant differences in mandibular length in OSAs patients compared to control groups. Stipa’s *et al*. (33), evaluated the correlation between AHI and cephalometric parameters, BMI, age, and gender, but no significant correlation was observed between AHI and Go-Me distance. On the contrary, Zucconi *et al*. (43) reported significant differences in mandibular length of OSAs patients compared to controls. Tepedino *et al*. (34) also observed a significant negative correlation between mandibular length (Co-Gn distance) and AHI, according to the results of the present study. Therefore, it was suggested that a shorter mandible could create an obstruction for airflow, potentially contributing to an increase in apnea episodes.

In the present study a negative correlation between maxilla length (Ans-Pns) and polysomnographic indices AHI and ODI, was observed. On the contrary Tepedino *et al*. (34) reported no significant correlation between AHI and maxilla length. Consistent with the findings of the present study, Seto *et al*. (44) and Sakabira *et al*. (45) demonstrated a decrease in maxilla length among OSAs patients compared to the control group. However, Lowe *et al*. (46) and Sforza *et al*. (47) observed no significant differences in maxilla length between the two groups. A decreased maxilla length is associated with an increase in obstructive events and blood desaturation. A reduction in maxillary and mandibular length could lead to a decrease in lingual space by pushing the tongue posteriorly, resulting in reduced pharyngeal patency.

A significant correlation between the cranial deflection angle and AHI and ODI was also observed in the present study. Many studies have analyzed changes in the skull base of OSAs patients (42,48) by studying the variations of cranial base angle (SNBa). Nevertheless, there are no studies that have analyzed the cranial deflection angle (Fh^NBa). It is unclear how the skull base could influence the pathogenesis of OSAs; variations in the deflection angle could potentially affect maxillary and mandibular positions and airway patency.

The present paper also demonstrated a significant correlation between the divergence angle and AHI: as the divergence angle increases, obstructive episodes and blood oxygen desaturation also increase. In patients with an increased angle of divergence, the mandible has a downward growth direction. When combined with altered posture, this could lead to a gradual narrowing of the airway, potentially promoting episodes of obstruction.

According to the present study, Cuccia *et al*. (49) showed an increase of divergence angle in OSAs patients compared to the control group. Similar findings were reported by Lowe *et al*. (50) and Laxmi *et al*. (51). Conversely, Stipa *et al*. (33) suggested that the divergence angle might not be a useful parameter for predicting AHI changes. Similar results were reported by Naughton *et al*. (35) for obese OSAs patients.

The findings of the current study reveal that a reduction in maxillomandibular length constitutes a risk factor for obstructive sleep apnea, as it leads to an increased frequency of apnea and hypopnea episodes as well as oxygen desaturations. Different studies have demonstrated the efficacy of maxillomandibular advancement in airway surgery for improving obstructive apnea. Considering that airway collapse can occur at various levels, different surgical approaches and techniques have been suggested to address obstructive apnea (52). Since anatomical changes can manifest in multiple area (including the oropharynx, nasopharynx, tongue, hyoid bone, maxilla, and mandible), each patient needs an individualized approach.

While there might be an anatomic predisposition in the pathogenesis of OSAs, non-anatomic factors also play a crucial role. These factors include the upper airway dilator muscles’ ability to respond to respiratory challenges during sleep, the tendency to awaken due to an increased respiratory drive during sleep (arousal threshold), and the stability of the respiratory control system (loop gain) (53).

Therefore, the present study suggested that maxilla and mandibular length, mandibular divergence and skull base alterations, in association with other variable factors, could influence OSAs pathogenesis, affecting OSAs indices.

The limitation of this study is due to the low value of rho coefficient despite having statistically significant correlation. Another limitation is related to the two-dimensional characteristics of the employed cephalometric examination, which was performed in an awake state and in an upright position. Due to the retrospective nature of this study it is difficult understand which other unanalyzed variables might have influenced the relationship between polysomnographic indices (AHI, ODI) and cephalometric parameters. Furthermore, because the analysed sample predominantly consisted of male patients, the associations observed between the polysomnographic indices and the cephalometric variables might have limited applicability to a female patient cohort.

The results of this study validate the impact of craniofacial morphology on the severity of obstructive sleep apnea. Clinicians are advised to thoroughly evaluate skeletal changes that contribute to OSAs and proactively initiate interventions for individuals at risk. This approach aims to alleviate the risks associated with obstructive sleep syndromes and preempt their early onset.

## Conclusions

In the present study, the authors suggested that certain alteration in craniofacial morphology, such as cranial deflection, mandibular divergence, and mandibular and maxillary length, could influence OSAs severity. The reduction in mandibular and maxillary length could lead to an increase in the number of apnoeic (AHI) and oxygen desaturation (ODI) events. The increase in the divergence angle could increase the apneic (AHI) events. The variations of cranial deflection angle could affect the number of the apnoeic (AHI) and of the oxygen desaturation (ODI) events.
